# Millimeter-Wave Radar-Based ECG Reconstruction Using Respiratory Harmonic Suppression and CA-WTBNet

**DOI:** 10.3390/bioengineering13070731

**Published:** 2026-06-24

**Authors:** Bowen Xiao, Chuyi Zhou, Lu Wang, Caiping Song, Yong Jia

**Affiliations:** 1 School of Mechanical and Electrical Engineering, Chengdu University of Technology, Chengdu 610059, China; 202320030123@stu.cdut.edu.cn (B.X.); 2024051450@stu.cdut.edu.cn (L.W.); 2022051227@stu.cdut.edu.cn (C.S.); 2 School of Information and Communication Engineering, University of Electronic Science and Technology of China, Chengdu 611731, China; 202522010327@std.uestc.edu.cn

**Keywords:** millimeter-wave radar, electrocardiogram reconstruction, vital signs, maximal overlap discrete wavelet transform, respiratory harmonic suppression, bidirectional long short-term memory, deep learning, non-contact cardiac monitoring, R-peak detection

## Abstract

Millimeter-wave radar enables non-contact monitoring of cardiac activity and therefore has the potential to reconstruct electrocardiogram signals without surface electrodes. However, existing radar-based electrocardiogram reconstruction methods still suffer from incomplete extraction of heartbeat-related information and insufficient modeling of electrocardiogram-related features, which limits reconstruction accuracy. To address these issues, this study proposes a millimeter-wave radar-based electrocardiogram reconstruction method that integrates a respiratory-harmonic-suppressed multi-channel signal-processing frontend with the proposed CA-WTBNet deep reconstruction network. First, based on maximal overlap discrete wavelet transform-based multi-resolution analysis, respiratory harmonics mixed into heartbeat-related components are suppressed by combining respiratory harmonic detection with a heart-rate frequency protection strategy, while cardiac-related information is preserved as much as possible. A multi-channel input representation is then constructed. Meanwhile, the proposed deep reconstruction network is developed to jointly model complementary channel-wise features, local waveform morphology, and temporal dependencies by integrating channel-attention mechanisms, convolutional residual modules, window-based Transformer blocks, and bidirectional long short-term memory. Experiments conducted on the public dataset show that our method achieves an average Pearson correlation coefficient of 0.9641, a mean normalized root mean square error of 0.0458, an average R-peak F1 score of 0.9956, and an average R-peak timing error of 3.13 ms on the test set. In comparison with related studies on the same public Resting dataset, the proposed method achieves the best overall performance among the compared methods, with a 0.53% improvement in Pearson correlation coefficient and a 10.20% reduction in normalized root mean square error over the best-performing compared method.

## 1. Introduction

Cardiovascular diseases (CVDs) have become a major global public health concern, causing 17.9 million deaths annually, and their incidence continues to rise [1]. Accurate monitoring of cardiac activity is essential for the prevention, diagnosis, and management of CVDs. As a non-invasive and reliable detection technique, electrocardiogram (ECG) has been widely used for the screening, diagnosis, and dynamic monitoring of cardiac diseases [1]. However, existing ECG devices generally rely on surface electrodes, which are susceptible to interference from skin conditions and sweat and present clear limitations for certain patient populations. For example, some patients are allergic to electrode materials [2], and electrodes may not be suitable for premature infants, burn patients, or patients with severe infections [3,4,5]. Millimeter-wave radar can sense millimeter-level displacement variations in the chest and abdomen caused by heartbeat and respiration. By analyzing the radar modulations induced by these displacement variations, cardiac activity can be inferred, enabling non-contact monitoring.

Traditional millimeter-wave radar methods for vital sign detection mainly focus on separating heartbeat components from radar echoes reflected by the human body and estimating simple parameters such as heart rate [6]. However, these methods can only output discrete statistical parameters, making it difficult to characterize dynamic variations in cardiac status over time. This limitation hinders the rapid, accurate, and comprehensive assessment of a patient’s cardiac health and may, in turn, affect subsequent diagnosis and treatment. To move beyond simple cardiopulmonary parameter estimation, radar-based vital-sign sensing, particularly millimeter-wave (mmWave) radar sensing, has gradually advanced toward the reconstruction of heartbeat waveforms and ECG signals [7,8,9].

**Figure 1:**
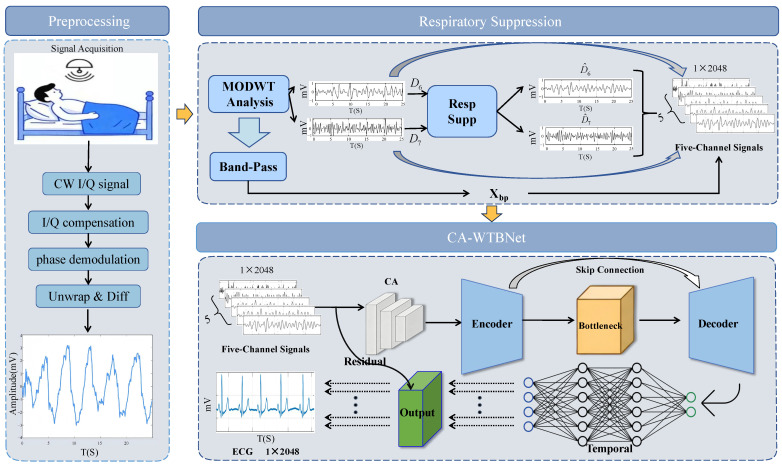
Framework of the proposed ECG reconstruction method, including preprocessing, respiratory-harmonic-suppressed multi-channel frontend construction, and CA-WTBNet-based ECG reconstruction.

This study proposes a millimeter-wave radar-based ECG reconstruction method that integrates a respiratory-harmonic-suppressed multi-channel signal processing frontend with a Channel-Attentive Windowed Transformer-BiLSTM Network (CA-WTBNet), as shown in Figure 1. First, based on MODWT multi-resolution analysis (MRA), respiratory harmonics mixed into heartbeat-related components are suppressed by combining respiratory harmonic detection with a heart-rate frequency protection strategy, while cardiac-related information is preserved as much as possible. A multi-channel input representation is then constructed. In addition, we develop CA-WTBNet as a deep reconstruction network. It combines channel attention, convolutional residual blocks, window-based Transformer modules, and bidirectional long short-term memory (BiLSTM) to model the multi-channel radar representation from multiple perspectives. With this design, the network can better capture complementary channel-wise information, local waveform morphology, and temporal dependencies. The main contributions of this study are summarized as follows.

First, we design a multi-channel frontend, which reduces respiration-related harmonics while preserving useful heartbeat information. Second, based on the proposed frontend, a CA-WTBNet reconstruction network is developed to improve the modeling of heartbeat-related features while keeping the model relatively lightweight. Third, a multi-objective loss function is designed to jointly constrain ECG reconstruction from three perspectives: waveform correlation, spectral consistency, and multi-scale temporal morphology. This helps improve the overall accuracy of the reconstructed ECG signals.

At the same time, this study is subject to several assumptions and limitations. The present study is limited to a public continuous-wave radar dataset comprising only healthy subjects in a resting state. Our primary objective was to evaluate the overall waveform reconstruction capability of the proposed radar-based method from an engineering and methodological perspective, rather than to establish a clinically validated diagnostic framework. Therefore, the proposed method is currently intended for controlled single-person recordings, and its performance in non-stationary scenarios, broader populations, and clinical settings still requires further validation.

The remainder of this paper is organized as follows. Section 2 reviews the related research and summarizes the existing methodological limitations. Section 3 formulates the problem addressed in this study together with the relevant assumptions and limitations. Section 4 presents the proposed method, including signal preprocessing, respiratory-harmonic-suppressed multi-channel frontend construction, and ECG reconstruction network design. Section 5 reports the experimental validation and comparative results. Section 6 discusses the main findings and outlines the open research questions. Finally, Section 7 concludes the paper.

## 2. Related Research

In recent years, advances in deep learning have enabled the reconstruction of intuitive ECG waveforms from radar signals. Using synchronously acquired heartbeat and ECG signals as training data, deep learning networks can learn the mapping from heartbeat signals to ECG signals [10,11]. To clarify the methodological context of this study, the related research is organized into two main routes, namely decomposition-based reconstruction and end-to-end reconstruction.

The first category extracts heartbeat components from radar signals and then reconstructs ECG signals using a dedicated reconstruction network. In [12], heartbeat signals were first separated from thoracic displacement signals using band-pass filtering and then used for ECG reconstruction through a hybrid deep learning model combining convolutional neural network (CNN) and long short-term memory (LSTM). Both [13,14] performed wavelet decomposition on preprocessed radar echo signals and selected four fixed wavelet coefficients as heartbeat components. The former applied a channel attention mechanism to the wavelet-decomposed signals and then reconstructed the ECG using a discriminative network composed of a convolutional neural network and gated recurrent unit (CNN-GRU). The latter first reconstructed heartbeat signals from four wavelet coefficients, extracted high-dimensional features through a convolutional autoencoder with an attention mechanism, recovered the signals using transposed convolutional layers, and finally used a bidirectional long short-term memory (BiLSTM) network for ECG reconstruction. In [15], WaveGRU-Net was proposed. It first applied fixed 5-level MODWT to decompose radar signals and fed all decomposed components into the network, then used a CNN-BiGRU model to learn temporal features and reconstruct complete ECG waveforms. In [16], the RBHHM method was proposed, which extracted heartbeat harmonics through bimodal Gaussian fitting and Shannon energy, clustered signals into three ECG-corresponding waves using K-means, and reconstructed ECG using recurrent neural networks (RNN).

The second category directly uses raw radar signals, their frequency spectra, or other derived mappings as network inputs to achieve end-to-end ECG reconstruction, while retaining complete heartbeat components and exploiting the coupling between respiration and heartbeat. However, the chest wall motion induced by heartbeat is smaller than that induced by respiration, and the second harmonic of respiration overlaps with the fundamental frequency of heartbeat [17,18,19]. As a result, subtle heartbeat features may be insufficiently emphasized during network learning. In [20,21], radar echo signals were converted into time–frequency spectrograms using short-time Fourier transform (STFT) and synchrosqueezing transform (SST), respectively, and then used as inputs to deep learning networks for end-to-end ECG reconstruction based on RSSRnet and the radarODE-MTL multitask learning framework. In [22], the study directly mapped ultra-wideband (UWB) radar signals to ECG waveforms by extracting amplitude and phase features and using a conditional generative adversarial network (GAN) with contrastive learning.

Overall, the reviewed studies indicate two main methodological limitations:

1. Heartbeat component extraction algorithms: Existing methods suffer from respiratory harmonic interference in heartbeat components. Without respiratory suppression, raw radar signals or fixed heartbeat components cannot retain cardiac details while eliminating interference. This distortion masks weak heartbeat features, impairs cardiac signal extraction and lowers ECG reconstruction accuracy.
2. ECG reconstruction networks: For decomposition-based ECG reconstruction methods, existing reconstruction networks still have limitations in jointly modeling the complementary information among different heartbeat components, local waveform features, and temporal dependencies. As a result, the extracted ECG micromotion features are often incomplete, which may degrade reconstruction performance.

## 3. Problem Statement

Against the background of related studies, the problem considered in this work can be stated as follows: how to sufficiently extract heartbeat-related information from radar signals affected by respiratory interference, and how to further improve the ECG reconstruction network. Radar echoes from the thoracic region carry both respiratory motion and heartbeat-related micromotion. Since respiratory harmonics can enter the heartbeat frequency range, the frontend should reduce this interference without removing useful cardiac information. Moreover, heartbeat-related information is very weak, easily disturbed by noise, and unevenly distributed across multiple channel components. In a deep learning network, these subtle details may be weakened or lost, so a mechanism is needed to help the model focus on the most relevant information. In addition, heartbeat-related signals contain temporal dependencies. Making better use of these dependencies is expected to improve the accuracy and temporal consistency of ECG reconstruction. Finally, a single-objective loss function is often insufficient for such a complex reconstruction task. It may improve one aspect of the reconstructed ECG while overlooking others, making it difficult to balance waveform similarity, spectral consistency, and temporal morphology simultaneously.

## 4. Materials and Methods

### 4.1. Preprocessing of Millimeter-Wave Radar Signals

**Figure 2:**
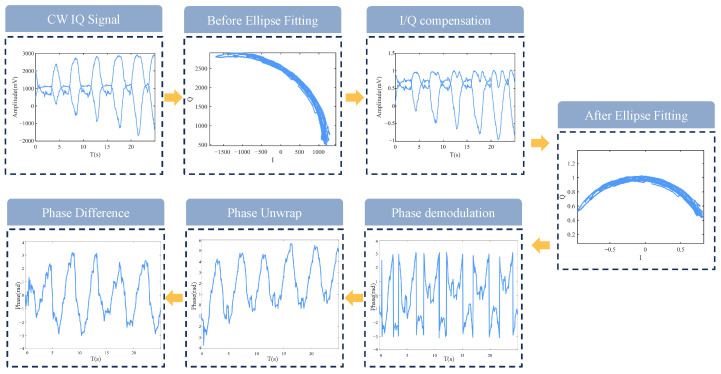
Flowchart of CW radar signal preprocessing.

In this study, experimental validation was conducted using a public dataset acquired with a custom-built 24GHz continuous-wave (CW) radar based on Six-Port technology [23]. In this dataset, the radar measurements are provided in the form of in-phase/quadrature (I/Q) signals. The preprocessing steps were tailored to the characteristics of CW radar I/Q signals. As illustrated in Figure 2, the pipeline begins with the initial quadrature-channel radar signals. The corresponding constellation plot of this initial signal is first displayed (pre-fit), followed by I/Q compensation, which is then visualized in a post-fit constellation plot to show the effect of the fitting. Subsequent phase processing steps include demodulation, unwrapping, and differencing. Specifically, the preprocessing pipeline includes I/Q signal extraction, I/Q signal compensation based on ellipse fitting to correct I/Q imbalance, DC offset, and quadrature distortion [24,25], phase demodulation using the arctangent operation, phase unwrapping to restore phase continuity, and phase differencing to suppress phase drift while enhancing the heartbeat-related component. The resulting differenced phase signal is then used as the input for the subsequent maximal overlap discrete wavelet transform-based multi-resolution analysis (MODWT-MRA) respiratory harmonic suppression frontend.

For CW radar, the received baseband signal is represented by the in-phase and quadrature (I/Q) channels, whose trajectories in the complex plane ideally form a circle. However, due to hardware imperfections, the measured I/Q data often exhibit an elliptical distribution. Therefore, ellipse-fitting-based compensation is performed before phase demodulation. The ellipse-fitting-based compensation procedure consists of three steps: translation, rotation, and scaling. First, the estimated center of the ellipse is subtracted from the measured I/Q data to remove the DC offset. Next, the compensated data are rotated according to the orientation of the ellipse principal axis so that the major and minor axes of the ellipse are aligned with the coordinate axes. Finally, axis-wise scaling is applied by stretching or compressing the data along the two principal axes, thereby transforming the ellipse into a circle. Through this process, channel gain imbalance and amplitude mismatch between the *I* and *Q* channels can be effectively compensated.

After I/Q compensation, phase demodulation is performed to extract the time-varying phase information associated with subtle physiological motion. Specifically, the four-quadrant arctangent function is applied to the selected radar signal to recover the phase trajectory [26]. By jointly utilizing the in-phase and quadrature components, this method enables more accurate estimation of phase variations and improves the integrity and stability of phase recovery, thereby providing a reliable basis for the subsequent extraction of heartbeat-related components. However, because the output range of the arctangent function is limited to [−π,π], phase discontinuities may arise, preventing the demodulated phase from faithfully reflecting the true temporal variation. To address this issue, phase unwrapping [27] is further performed. Specifically, when the phase difference between two adjacent samples exceeds π, the phase is corrected by ±2π, thereby yielding the continuous unwrapped phase signal, denoted as $\phi_{w}\left[n\right]$.

Based on the unwrapped phase, phase differencing is then applied to further suppress low-frequency phase drift and enhance the heartbeat-related component. By calculating the phase difference between adjacent discrete samples, slowly varying trends can be effectively attenuated, while subtle variations induced by cardiac motion are emphasized. The resulting differenced phase signal, denoted by $\phi_{e}\left[n\right]$, serves as the key input to the subsequent Respiratory Suppression stage.

### 4.2. MODWT-MRA-Based Respiration-Synchronized Harmonic Suppression Frontend

In radar-based vital sign signal processing, the discrete wavelet transform (DWT) provides multi-scale analysis with relatively low computational complexity. Combined with the Mallat algorithm [28], DWT enables fast decomposition and is therefore more suitable for real-time monitoring than variational mode decomposition (VMD) [29]. However, conventional DWT relies on downsampling, which may introduce translation sensitivity and frequency-band aliasing. To address this limitation, the maximal overlap discrete wavelet transform (MODWT) was introduced by Percival and Walden [30]. Since MODWT avoids downsampling, it improves translation invariance and reduces information loss, making it more suitable for extracting heartbeat-related components from non-stationary radar signals.

**Figure 3:**
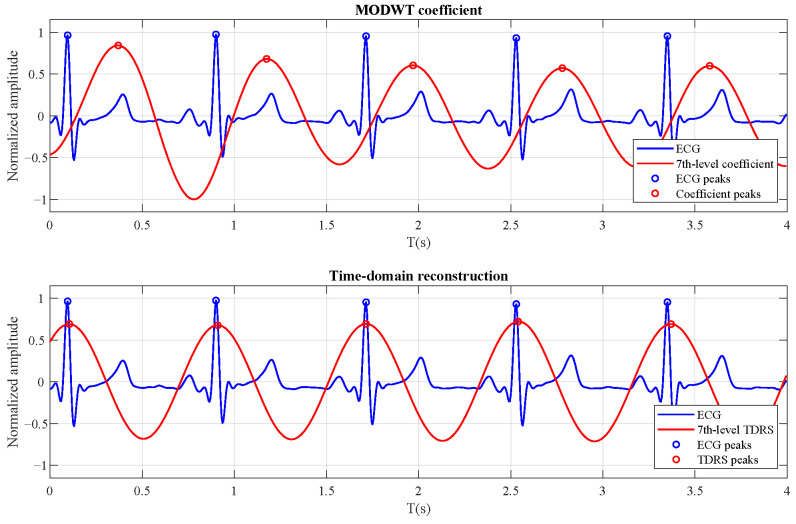
Comparison of the temporal alignment between the selected MODWT coefficients, their corresponding time-domain reconstructed signals (TDRSs), and the reference ECG signal.

The MODWT decomposition adopts the sym4 wavelet basis in this study, owing to its favorable orthogonality, near-symmetry, and phase-preserving properties, which are beneficial for reducing phase distortion and retaining transient ECG-related features [31,32]. Furthermore, instead of directly using MODWT coefficients, MODWT-based multiresolution analysis (MODWT-MRA) is employed to reconstruct level-specific time-domain components. These MRA-derived components preserve the additive decomposition property and provide better temporal alignment with the original radar signal, which is important for ECG waveform reconstruction. As shown in Figure 3, compared with the selected MODWT coefficients, the corresponding time-domain reconstructed signals (TDRSs) exhibit better temporal consistency with the reference ECG waveform. Therefore, the MRA-derived TDRSs are used as the cardiac-related inputs for the subsequent ECG reconstruction network. The proposed frontend consists of four main steps.

1. MODWT-MRA decomposition and cardiac-related component selection.
  For the radar phase signal $x\in R^{L}$ output from the preprocessing stage, we first apply MODWT-MRA to decompose it into level-specific time-domain components, as expressed in Equation (1):
  (1) $$D_{\ell }={\mathrm{MRA}}_{\ell }\left(\mathrm{MODWT}\left(x,\psi_{\mathrm{sym}4},J\right)\right),\;\ell =1,2,\ldots ,J.$$
  where MODWT(·) denotes the MODWT decomposition operator, ${\mathrm{MRA}}_{\ell }\left(\cdot \right)$ denotes the MRA reconstruction operation for the *ℓ*-th level, $\psi_{\mathrm{sym}4}$ is the sym4 wavelet basis, and J=10 is the decomposition level. Thus, $D_{\ell }\in R^{L}$ represents the time-domain reconstructed component corresponding to the *ℓ*-th MODWT level. In this study, the sampling frequency is $f_{s}=200\;\mathrm{Hz}$ and the segment length is L=2048. According to the heartbeat-related frequency distribution, $D_{6}$ and $D_{7}$ are selected as the main cardiac-dominant MRA components. In addition, the original radar signal is band-pass filtered within 0.8–2.0Hz to obtain an auxiliary cardiac-band component $x_{\mathrm{bp}}$.
2. Respiration-synchronized harmonic identification.
  The respiratory reference is extracted from the original radar phase signal within 0.1–0.5Hz, and the respiratory fundamental frequency $f_{r}$ is estimated as the dominant spectral peak in this band. Respiratory harmonic candidates are then searched within the cardiac frequency band $B_{c}=\left[0.8,2.0\right]\;\mathrm{Hz}$. For the *k*-th candidate harmonic, the harmonic frequency $f_{h,k}$ and the harmonic half-bandwidth $\Delta_{h}$ are defined in Equation (2):
  (2) $$f_{h,k}=kf_{r},\;\Delta_{h}=\min\left(0.035,0.20f_{r}\right),$$
  where $f_{h,k}$ is the candidate harmonic frequency and $\Delta_{h}$ is the corresponding harmonic half-bandwidth. To determine whether a candidate frequency is truly synchronized with respiration, both spectral prominence and respiration-synchronization constraints are considered. For each candidate $f_{h,k}$, $E_{h,k}$ is the spectral energy of $D_{6}+D_{7}$ within $\left[f_{h,k}-\Delta_{h},f_{h,k}+\Delta_{h}\right]$, while $E_{bg,k}$ is the background energy in the neighboring region, excluding this harmonic band. This energy measure acts as a common spectral-prominence constraint for both selected MRA components. For each selected component $D_{\ell }$, ℓ∈{6,7}, the band-limited part around $f_{h,k}$, called $z_{\ell ,k}\left(t\right)$, is fitted using the respiratory harmonic phase. The corresponding regression model is expressed in Equation (3):
  (3) $$z_{\ell ,k}\left(t\right)\approx a_{\ell ,k}\cos\left(k\phi_{r}\left(t\right)\right)+b_{\ell ,k}\sin\left(k\phi_{r}\left(t\right)\right)+c_{\ell ,k},$$
  where $\phi_{r}\left(t\right)$ is the instantaneous respiratory phase, and $a_{\ell ,k}$, $b_{\ell ,k}$, and $c_{\ell ,k}$ are regression coefficients. The regression-based correlation score $R_{\ell ,k}$ is calculated from the fitting quality, and a maximum lag range of ±0.40s is used to tolerate possible phase delay. To further measure the synchronization between the candidate harmonic and the respiratory phase, the phase-locking value ${\mathrm{PLV}}_{\ell ,k}$ is obtained as shown in Equation (4):
  (4) $${\mathrm{PLV}}_{\ell ,k}=\left|\frac{1}{L}\sum_{t=1}^{L}\exp\left[j\left(\phi_{\ell ,k}\left(t\right)-k\phi_{r}\left(t\right)\right)\right]\right|,$$
  where $\phi_{\ell ,k}\left(t\right)$ denotes the instantaneous phase of $z_{\ell ,k}\left(t\right)$ and *j* is the imaginary unit.
  Then, for each selected MRA component $D_{\ell }$, ℓ∈{6,7}, Equation (5) is used separately to check whether each candidate frequency belongs to the component-specific respiration-synchronized harmonic set $H_{\ell }$.
  (5) $$f_{h,k}\in H_{\ell }\;\mathrm{if}\;E_{h,k}>\gamma_{h}E_{bg,k}\;\mathrm{and}\;\left(R_{\ell ,k}\geq \tau_{R}\;\mathrm{or}\;{\mathrm{PLV}}_{\ell ,k}\geq \tau_{\mathrm{PLV}}\right),\;\ell \in \left\{6,7\right\}.$$
  Here, $H_{\ell }$ is the set of identified respiration-synchronized harmonics for the current MRA component $D_{\ell }$. $R_{\ell ,k}$ and ${\mathrm{PLV}}_{\ell ,k}$ are the regression-based synchronization coefficient and phase-locking value between $D_{\ell }$ and the respiratory harmonic phase. Thus, the harmonic detection and suppression are performed separately for $D_{6}$ and $D_{7}$. In the default setting, $\gamma_{h}=1.6$, $\tau_{R}=0.20$, and $\tau_{\mathrm{PLV}}=0.45$.
3. Cardiac-frequency protection before harmonic suppression.
  True cardiac components may be close to respiratory harmonics, so suppressing all confirmed harmonics is not always safe. It may also weaken useful heartbeat-related information. To reduce this risk, possible cardiac frequencies are estimated before the suppression step. In particular, the spectrum of $D_{6}+D_{7}$ is searched in the range 0.8–2.0Hz and local spectral peaks are identified in this band. The two peaks with the highest spectral energy are kept under a minimum peak distance of 0.12Hz, forming the cardiac-frequency candidate set $F_{\mathrm{card}}$. Equation (6) is then used as the protection rule: a confirmed harmonic $f_{h,k}\in H_{\ell }$ is treated as protected if it lies sufficiently close to any frequency in $F_{\mathrm{card}}$:
  (6) $$\min_{f_{c}\in F_{\mathrm{card}}}\left|f_{h,k}-f_{c}\right|<\Delta_{\mathrm{card}}.$$
  where $\Delta_{\mathrm{card}}=0.12\;\mathrm{Hz}$ is the cardiac-frequency protection bandwidth. Protected harmonics are therefore only slightly suppressed, whereas non-protected harmonics are suppressed more strongly.
4. Frequency-domain soft suppression and five-channel frontend construction.
  For each confirmed harmonic in $H_{\ell }$, frequency-domain Gaussian soft suppression is applied to the corresponding component $D_{\ell }$, where ℓ∈{6,7}. The suppression mask is defined in Equation (7).
  (7) $$M_{\ell ,k}\left(f\right)=1-\alpha_{\ell ,k}\exp\left[-\frac{{\left(f-f_{h,k}\right)}^{2}}{2\sigma_{h}^{2}}\right],\;\ell \in \left\{6,7\right\}.$$
  where $\sigma_{h}=\eta_{\sigma }\Delta_{h}$ controls the width of the Gaussian attenuation window and $\eta_{\sigma }=0.85$ is used in the default setting. The suppression strength $\alpha_{\ell ,k}$ is estimated from the respiration-synchronization score $S_{\ell ,k}=\max\left(R_{\ell ,k},{\mathrm{PLV}}_{\ell ,k}\right)$. Non-protected harmonics are more strongly weakened with a suppression gain of 0.90 and $\alpha_{\ell ,k}$ clipped to 0.45–0.85. On the other hand, protected harmonics are attenuated only weakly. For these harmonics, the suppression gain is decreased to 0.15 and $\alpha_{\ell ,k}$ is restricted to 0.02–0.12. The mask is then applied symmetrically to the positive and negative frequency components of the corresponding component $D_{\ell }$. The inverse Fourier transform is then applied to obtain the respiration-attenuated components ${\hat{D}}_{6}$ and ${\hat{D}}_{7}$.
  Finally, in Equation (8), the original cardiac-dominant components, the respiration-attenuated complementary components, and the auxiliary cardiac-band component are concatenated to form the five-channel frontend output $X_{\mathrm{raw}}$:
  (8) $$X_{\mathrm{raw}}=\left[D_{6};D_{7};{\hat{D}}_{6};{\hat{D}}_{7};x_{\mathrm{bp}}\right]\in R^{5\times L}.$$
  The original components $D_{6}$ and $D_{7}$ preserve unmodified cardiac-dominant information, while ${\hat{D}}_{6}$ and ${\hat{D}}_{7}$ provide respiration-attenuated complementary representations. The five-channel representation $X_{\mathrm{raw}}$ is then fed into the subsequent ECG reconstruction network.

### 4.3. ECG Reconstruction Based on CA-WTBNet

**Figure 4:**
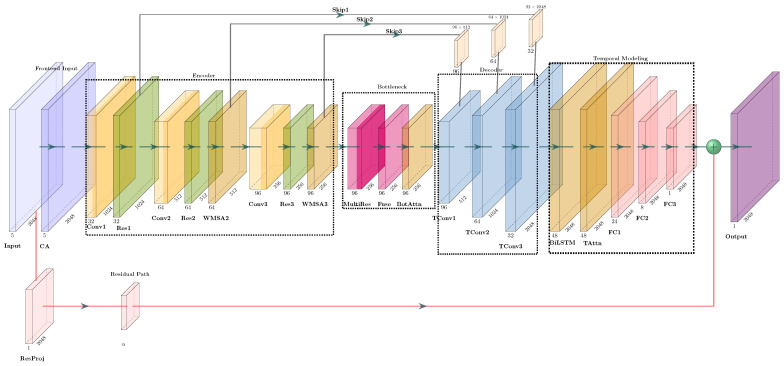
Architecture of the proposed CA-WTBNet.

The architecture of the proposed backend reconstruction network is illustrated in Figure 4. The network is termed CA-WTBNet, which stands for Channel-Attentive Windowed Transformer-BiLSTM Network. It consists of an input channel attention module, a convolutional encoder with window-based Transformer blocks, a bottleneck feature enhancement module, a decoder with skip connections, and a temporal modeling and output reconstruction module based on BiLSTM.

#### 4.3.1. Channel Attention Module

**Figure 5:**
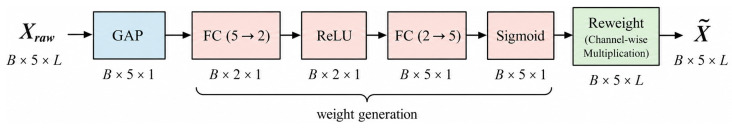
Structure of the input channel attention module in CA-WTBNet.

After the multi-channel frontend representation is constructed, heartbeat-related information is not uniformly distributed across different input channels. Therefore, an equal-weight fusion strategy may be insufficient to distinguish the relative importance of different components. To address this issue, a channel attention (CA) module is designed based on the Squeeze-and-Excitation Network (SENet) mechanism [33], which adaptively assigns different weights to different input channels. The structure of the CA module is illustrated in Figure 5. This module enhances channels containing more informative heartbeat-related components, while further suppressing redundant channels affected by residual respiration, motion artifacts, and noise. Let the raw frontend input be denoted as $X_{\mathrm{raw}}\in R^{B\times C_{\mathrm{in}}\times L}$, where *B* is the batch size, $C_{\mathrm{in}}$ is the number of input channels, and *L* is the temporal length. In this study, $C_{\mathrm{in}}=5$, corresponding to $D_{6}$, $D_{7}$, ${\hat{D}}_{6}$, ${\hat{D}}_{7}$, and $x_{\mathrm{bp}}$. The channel attention weights are generated by Equation (9).

(9) $$\omega_{\mathrm{ca}}=\sigma \left(W_{\mathrm{ca},2}\;\mathrm{ReLU}\left(W_{\mathrm{ca},1}\;\mathrm{GAP}\left(X_{\mathrm{raw}}\right)+b_{\mathrm{ca},1}\right)+b_{\mathrm{ca},2}\right),$$

where GAP(·) denotes global average pooling along the temporal dimension, $W_{\mathrm{ca},1}$ and $W_{\mathrm{ca},2}$ are learnable weight matrices, $b_{\mathrm{ca},1}$ and $b_{\mathrm{ca},2}$ are bias terms, and σ(·) is the sigmoid function. The recalibrated input to the encoder is then obtained using Equation (10).

(10) $$\tilde{X}=\omega_{\mathrm{ca}}⊙X_{\mathrm{raw}},$$

where ⊙ denotes channel-wise multiplication with broadcasting along the temporal dimension. The resulting $\tilde{X}$ is used as the encoder input, allowing the network to emphasize informative ECG-related channels while suppressing redundant or noise-dominated components.

#### 4.3.2. Encoder Module

**Figure 6:**
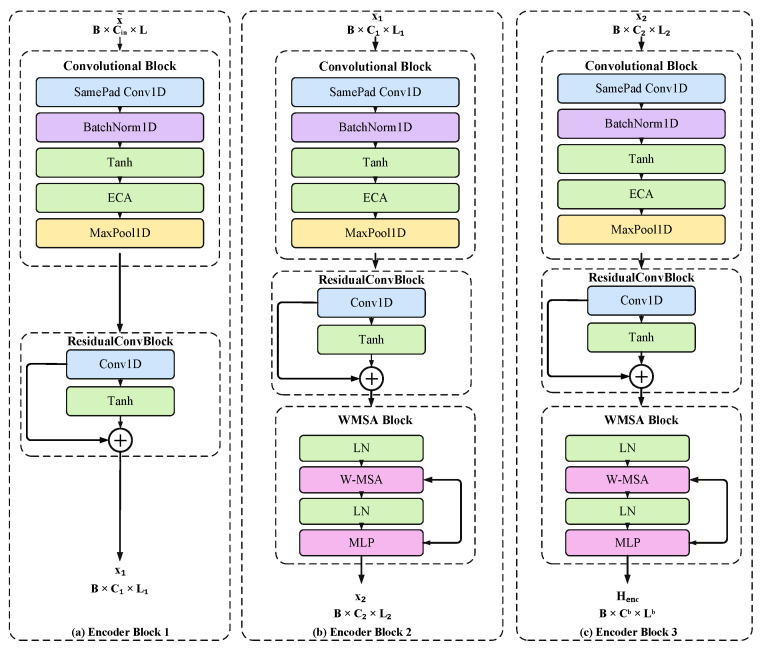
Internal structures of the encoder blocks in CA-WTBNet.

The encoder is designed to extract hierarchical temporal representations from the multi-channel mmWave radar features. Let the output of the input channel attention module be denoted as $\tilde{X}\in R^{B\times C_{\mathrm{in}}\times L}$, where *B* is the batch size, $C_{\mathrm{in}}$ is the number of input channels, and *L* is the temporal length. As shown in Figure 6, the encoder consists of three convolutional encoding stages. Each stage contains a convolutional block and a residual convolutional block, while window-based Transformer blocks are inserted after the second and third stages to strengthen temporal dependency modeling.

Set $x_{0}=\tilde{X}$. The encoder gradually transforms the input representation through three hierarchical stages as described in Equation (11)–(13). The first stage mainly extracts local waveform features and the second and third stages further introduce window-based Transformer blocks to enhance the temporal dependency modeling:

(11) $$x_{1}={\mathrm{ResBlock}}_{1}\left({\mathrm{ConvBlock}}_{1}\left(x_{0}\right)\right),$$

(12) $$x_{2}={\mathrm{TransformerBlock}}_{\mathrm{enc}2}\left({\mathrm{ResBlock}}_{2}\left({\mathrm{ConvBlock}}_{2}\left(x_{1}\right)\right)\right),$$

(13) $$H_{\mathrm{enc}}={\mathrm{TransformerBlock}}_{\mathrm{enc}3}\left({\mathrm{ResBlock}}_{3}\left({\mathrm{ConvBlock}}_{3}\left(x_{2}\right)\right)\right).$$

Here, $x_{1}\in R^{B\times C_{1}\times L_{1}}$ and $x_{2}\in R^{B\times C_{2}\times L_{2}}$ denote the intermediate encoder features, while $H_{\mathrm{enc}}\in R^{B\times C_{b}\times L_{b}}$ denotes the final encoder representation. $C_{1}$, $C_{2}$, and $C_{b}$ represent the channel numbers at different encoding stages, and $L_{1}$, $L_{2}$, and $L_{b}$ denote the corresponding temporal lengths.

Each convolutional block is used to perform local waveform feature extraction and downsampling. Specifically, a same-padded one-dimensional convolution [34] is followed by batch normalization [35], tanh activation, Efficient Channel Attention (ECA)-based channel recalibration, and max-pooling. For an input feature h, the ECA operation is defined as Equation (14).

(14) $$\mathrm{ECA}\left(h\right)=\sigma \left(\mathrm{Conv}1D_{k}\left(\mathrm{GAP}\left(h\right)\right)\right)⊙h,$$

where GAP(·) denotes global average pooling along the temporal dimension, $\mathrm{Conv}1D_{k}\left(\cdot \right)$ denotes a lightweight one-dimensional convolution with kernel size *k* for modeling local cross-channel interactions, σ(·) is the sigmoid function, and ⊙ denotes channel-wise multiplication. Following ECA-Net [36], the kernel size *k* is adaptively determined by the channel number *C* as Equation (15).

(15) $$k=\psi \left(C\right)={\left|\frac{\log_{2}\left(C\right)}{\gamma }+\frac{b}{\gamma }\right|}_{\mathrm{odd}},$$

where ${\left|\cdot \right|}_{\mathrm{odd}}$ denotes the nearest odd integer, and γ and *b* are set to 2 and 1, respectively. Through this operation, the convolutional block can adaptively emphasize informative feature channels and suppress redundant responses.

The residual convolutional block is further introduced after each convolutional block to refine the extracted temporal features. It uses residual learning to preserve the input information while enhancing local morphological patterns. This design helps to stabilize feature propagation and improves the representation of weak heartbeat-related components in non-stationary radar signals.

To enhance the encoder’s ability to capture long-range temporal dependencies, we incorporate a self-attention mechanism. The window-based multi-head self-attention (W-MSA) design is inspired by efficient Transformer architectures such as Swin Transformer [37]. Rather than applying full-sequence global attention, which can be computationally heavy, the W-MSA operates within local windows. This approach allows the model to efficiently capture temporal relationships inside each window while keeping the computational cost manageable. Accordingly, W-MSA is applied to the intermediate and high-level encoder features. For an input feature h, the attention sublayer is formulated as Equation (16).

(16) $$h^{\prime }=h+\rho_{\mathrm{att}}\;W-\mathrm{MSA}\left(\mathrm{LayerNorm}\left(h\right)\right),$$

Here, $\rho_{\mathrm{att}}$ is a learnable residual scaling factor and W−MSA(·) denotes window-based multi-head self-attention. Specifically, within each local temporal window, the W-MSA operation is computed as Equation (17).

(17) $$W-\mathrm{MSA}\left(Q,K,V\right)=\mathrm{softmax}\left(\frac{Q_{w}K_{w}^{T}}{\sqrt{d_{k}}}+M\right)V_{w},$$

where $Q_{w}$, $K_{w}$ and $V_{w}$ refer to the query, key, and value matrices in a local window, respectively; $d_{k}$ is the dimension of the key vectors; and M is the attention mask used to confine the attention computation within the corresponding window. This enables the model to attend to temporal relationships without the high computational cost of self-attention over the entire sequence.

The feed-forward network (FFN) sublayer is then given by Equation (18).

(18) $$\mathrm{TransformerBlock}\left(h\right)=h^{\prime }+\rho_{\mathrm{ffn}}\;\mathrm{FFN}\left(\mathrm{LayerNorm}\left(h^{\prime }\right)\right),$$

where $\rho_{\mathrm{ffn}}$ is another learnable residual scaling factor. Here, FFN(·) denotes the feed-forward network and LayerNorm(·) denotes layer normalization.

The encoder progressively reduces the temporal resolution via successive convolution, residual refinement, and window-based self-attention, and enhances the feature representation capacity. The convolutional blocks are designed to learn the local patterns of the heartbeat. The residual convolutional blocks improve the learned features. Furthermore, the window-based Transformer blocks are used to learn the long-range dependencies more effectively. Overall, the encoder produces an efficient and ECG-informative representation for the subsequent bottleneck feature enhancement module.

#### 4.3.3. Bottleneck Feature Enhancement Module

After the encoder, a bottleneck feature enhancement module is introduced to further refine the compressed high-level representation before temporal resolution recovery. Let the encoder output be denoted as $H_{\mathrm{enc}}\in R^{B\times C_{b}\times L_{b}}$, where *B* is the batch size, $C_{b}$ is the number of bottleneck feature channels, and $L_{b}$ is the reduced temporal length. At this stage, the temporal resolution has been reduced, while the channel dimension has been increased. Therefore, the bottleneck module is designed to enhance the compact feature representation by combining multi-scale convolutional feature extraction and attention-based dependency modeling.

First, a multi-resolution convolutional branch is applied to $H_{\mathrm{enc}}$. This branch contains several parallel one-dimensional convolutional paths with different kernel sizes. These parallel paths allow the network to capture temporal patterns over different receptive-field scales. In this study, the kernel set is denoted as K. The multi-resolution feature extraction process is formulated as Equation (19).

(19) $$\mathrm{MultiRes}\left(H_{\mathrm{enc}}\right)=\mathrm{Mix}\left({\left[{\mathrm{Branch}}_{k}\left(H_{\mathrm{enc}}\right)\right]}_{k\in K}\right),$$

where ${\mathrm{Branch}}_{k}\left(\cdot \right)$ denotes a one-dimensional convolutional branch with kernel size *k*, ${\left[\cdot \right]}_{k\in K}$ denotes the channel-wise concatenation of features extracted by all branches, and Mix(·) denotes a 1×1 convolution used for feature fusion. Through the parallel convolutional branches, the module can simultaneously capture short-term local morphological variations and broader temporal patterns in the compressed feature space.

The multi-resolution feature is then added back to the original encoder output through a residual connection. This residual fusion preserves the original high-level representation while introducing multi-scale temporal information. As described in Equation (20), the fused feature is further processed by a bottleneck attention module to obtain the bottleneck representation:

(20) $$H_{\mathrm{bot}}=\mathrm{BottleneckAttn}\left(H_{\mathrm{enc}}+\mathrm{MultiRes}\left(H_{\mathrm{enc}}\right)\right).$$

Here, $H_{\mathrm{bot}}\in R^{B\times C_{b}\times L_{b}}$ denotes the enhanced bottleneck representation and BottleneckAttn(·) denotes the attention operation applied in the bottleneck feature space. Since this attention module operates on the compressed temporal representation, it can model long-range dependencies with a moderate computational cost.

Through this design, the bottleneck module further strengthens the encoder output prior to temporal resolution recovery. The multi-resolution branch enriches the feature representation at different temporal scales, whereas the bottleneck attention module improves the modeling of global temporal dependencies. The enhanced representation $H_{\mathrm{bot}}$ is then passed to the decoder for temporal resolution recovery.

#### 4.3.4. Decoder Module

The decoder is used to gradually recover the temporal resolution from the compressed bottleneck representation and generate features for ECG waveform reconstruction. Let the bottleneck output be denoted as $H_{\mathrm{bot}}\in R^{B\times C_{b}\times L_{b}}$, where *B* is the batch size, $C_{b}$ is the number of bottleneck feature channels, and $L_{b}$ is the reduced temporal length. The decoder consists of three transposed-convolution stages, which progressively upsample the feature map along the temporal dimension.

To preserve multi-level temporal information learned by the encoder, skip connections are introduced between the encoder and decoder. Specifically, the final encoder output and two intermediate encoder features are reused during decoding. Let $z_{1}=H_{\mathrm{enc}}$, $z_{2}=x_{2}$, and $z_{3}=x_{1}$, where $H_{\mathrm{enc}}$, $x_{2}$, and $x_{1}$ represent the deep, intermediate, and shallow encoder features, respectively. For each decoding stage, the corresponding skip feature is first temporally aligned by linear interpolation and then projected by a 1×1 convolution to match the channel dimension of the decoder feature. The decoding process is formulated as Equations (21)–(23).

(21) $$d_{0}=H_{\mathrm{bot}},$$

(22) $$d_{i}={\mathrm{TransConv}}_{i}\left(d_{i-1}\right)+s_{i}{\mathrm{Conv}}_{1\times 1}^{\left(i\right)}\left(\mathrm{Interp}\left(z_{i}\right)\right),\;i=1,2,3,$$

(23) $$H_{\mathrm{dec}}=d_{3}.$$

Here, ${\mathrm{TransConv}}_{i}\left(\cdot \right)$ denotes the *i*-th transposed-convolution block, Interp(·) denotes linear interpolation used for temporal alignment, and ${\mathrm{Conv}}_{1\times 1}^{\left(i\right)}\left(\cdot \right)$ denotes the 1×1 convolution used for channel matching before skip fusion. The coefficient $s_{i}$ is a learnable skip-scaling factor that controls the contribution of the corresponding encoder feature. The three skip paths correspond to Skip Conv3, Skip Conv2, and Skip Conv1 respectively.

Through progressive transposed convolution and multi-level skip fusion, the decoder restores the temporal resolution while retaining encoder features at different temporal scales. The deep skip feature provides compact high-level information, whereas the intermediate and shallow skip features help preserve local ECG morphological details. In this way, the decoder provides temporally recovered and morphology-aware features for the subsequent BiLSTM-based reconstruction module.

#### 4.3.5. Temporal Modeling and Output Reconstruction Module

After the decoder, a temporal modeling and output reconstruction module is used to generate the final ECG waveform. Let the decoder output be denoted as $H_{\mathrm{dec}}\in R^{B\times C_{d}\times L}$, where *B* is the batch size, $C_{d}$ is the number of decoder output channels, and *L* is the temporal length. The feature map is rearranged along the temporal dimension into a sequence matrix $U={\left[u_{1},u_{2},\ldots ,u_{L}\right]}^{T}\in R^{L\times C_{d}}$, where $u_{t}\in R^{C_{d}}$ represents the feature vector at the *t*-th time step.

The morphology and duration of individual ECG waveform components exhibit bidirectional dependencies. Specifically, in ECG signals, the waveform features at a given time step are associated not only with preceding cardiac electrical activity, but also with subsequent cardiac electrical activity [38]. LSTM is a recurrent neural network architecture for sequential data processing that includes three gates, namely the input gate, forget gate, and output gate, along with a cell state [39]. BiLSTM is a bidirectional extension of LSTM [40]. Therefore, we begin by using BiLSTM to model temporal dependencies in the forward ${\vec{h}}_{t}$ direction and the backward direction ${\overset{\leftarrow }{h}}_{t}$. The bidirectional temporal representation is given by Equation (24).

(24) $$H^{\mathrm{lstm}}={\left[h_{1}^{\mathrm{lstm}},h_{2}^{\mathrm{lstm}},\ldots ,h_{L}^{\mathrm{lstm}}\right]}^{T}=\mathrm{BiLSTM}\left(U\right),\;h_{t}^{\mathrm{lstm}}=\left[{\vec{h}}_{t};{\overset{\leftarrow }{h}}_{t}\right],\;t=1,\ldots ,L.$$

By using bidirectional recurrent modeling, the network can exploit temporal context from both past and future time steps, which is beneficial for maintaining the continuity of the reconstructed ECG waveform.

To further emphasize ECG-informative time positions, a temporal attention module shown in Equation (25) is applied to the BiLSTM output:

(25) $${\tilde{h}}_{t}^{\mathrm{lstm}}=\alpha_{t}h_{t}^{\mathrm{lstm}},\;\alpha_{t}=\sigma \left(w_{2}^{T}\tanh\left(W_{1}h_{t}^{\mathrm{lstm}}\right)\right).$$

where $\alpha_{t}$ is the attention weight at the *t*-th time step and $W_{1}$ and $w_{2}$ are learnable parameters. This operation allows the model to assign larger weights to time positions that contain more useful ECG-related information.

The attention-weighted features are then fed into a time-distributed fully connected mapping module to produce an initial ECG reconstruction ${\hat{y}}_{\mathrm{FC}}$. This module maps each temporal feature vector to one ECG amplitude value, and the outputs over all time steps form the reconstructed waveform. The mapping module consists of three fully connected layers with nonlinear activation and dropout between adjacent layers.

Finally, an input-to-output residual connection is introduced to preserve low-level temporal information from the original frontend input. The final reconstructed ECG waveform is defined as Equation (26).

(26) $$\hat{y}={\hat{y}}_{\mathrm{FC}}+\lambda \cdot {\mathrm{Conv}}_{1\times 1}\left(X_{\mathrm{raw}}\right).$$

Here, $\hat{y}$ denotes the final reconstructed ECG signal, $X_{\mathrm{raw}}$ denotes the original multi-channel frontend input, ${\mathrm{Conv}}_{1\times 1}\left(\cdot \right)$ projects the input feature to a single-channel residual component, and λ is a learnable residual scaling factor.

Overall, this module combines BiLSTM-based temporal modeling, temporal attention, fully connected amplitude mapping, and residual output fusion. It enables the network to capture long-range temporal dependencies while preserving useful low-level information, thereby improving the temporal consistency and morphological stability of the reconstructed ECG waveform.

#### 4.3.6. Multi-Objective Loss Function

To improve the quality of ECG reconstruction, this study adopts a multi-objective loss function to jointly optimize amplitude accuracy, waveform correlation, spectral consistency, and multi-scale morphological similarity. The total loss is defined as Equation (27).

(27) $$L=w_{\mathrm{huber}}L_{\mathrm{huber}}+w_{\mathrm{pcc}}L_{\mathrm{pcc}}+w_{\mathrm{freq}}L_{\mathrm{freq}}+w_{\mathrm{mspcc}}L_{\mathrm{mspcc}},$$

The corresponding weights were empirically set to $w_{\mathrm{huber}}=1.0$, $w_{\mathrm{pcc}}=3.0$, $w_{\mathrm{freq}}=0.02$, and $w_{\mathrm{mspcc}}=0.05$.

The Huber loss is used as the basic point-wise reconstruction loss, which is calculated in Equation (28):

(28) $$L_{\mathrm{huber}}=\frac{1}{N}\sum_{i=1}^{N}\left\{\begin{array}{cc} \frac{1}{2}{\left({\hat{y}}_{i}-y_{i}\right)}^{2},\; & \left|{\hat{y}}_{i}-y_{i}\right|\leq \delta ,\\ \delta \left|{\hat{y}}_{i}-y_{i}\right|-\frac{1}{2}\delta^{2},\; & \mathrm{otherwise}, \end{array}\right.$$

where *N* denotes the number of ECG samples used in the loss calculation, ${\hat{y}}_{i}$ and $y_{i}$ denote the reconstructed and reference ECG samples, respectively, and δ=0.08.

The PCC loss in Equation (29) is used to enhance the global waveform correlation between the reconstructed and reference ECG signals:

(29) $$L_{\mathrm{pcc}}=1-\frac{1}{B}\sum_{b=1}^{B}\frac{{\left({\hat{y}}_{b}-{\bar{\hat{y}}}_{b}\right)}^{T}\left(y_{b}-{\bar{y}}_{b}\right)}{{\left\|{\hat{y}}_{b}-{\bar{\hat{y}}}_{b}\right\|}_{2}{\left\|y_{b}-{\bar{y}}_{b}\right\|}_{2}+\epsilon },$$

where *B* denotes the batch size, ${\hat{y}}_{b}$ and $y_{b}$ denote the reconstructed and reference ECG segment of the *b*-th sample, respectively, ${\bar{\hat{y}}}_{b}$ and ${\bar{y}}_{b}$ are their temporal mean values, ${∥\cdot ∥}_{2}$ denotes the Euclidean norm, and ϵ is a small constant used for numerical stability.

The frequency-domain loss in Equation (30) is then introduced using the fast Fourier transform (FFT) to preserve spectral consistency between the reconstructed and reference ECG signals:

(30) $$L_{\mathrm{freq}}=\mathrm{SmoothL}1\left(\log\left(1+\left|\mathrm{FFT}\left(\hat{y}\right)\right|\right),\log\left(1+\left|\mathrm{FFT}\left(y\right)\right|\right)\right).$$

Furthermore, a multi-scale PCC loss shown in Equation (31) is used to constrain waveform similarity at different temporal resolutions:

(31) $$L_{\mathrm{mspcc}}=\frac{1}{4}\left[L_{\mathrm{pcc}}\left(\hat{y},y\right)+\sum_{s\in \{2,4,8\}}L_{\mathrm{pcc}}\left({\mathrm{AvgPool}}_{s}\left(\hat{y}\right),{\mathrm{AvgPool}}_{s}\left(y\right)\right)\right],$$

where ${\mathrm{AvgPool}}_{s}\left(\cdot \right)$ denotes average pooling with kernel size and stride equal to *s*. This multi-objective design encourages the model to reconstruct ECG signals with accurate amplitudes, high waveform correlation, consistent spectral characteristics, and stable morphology across multiple temporal scales.

### 4.4. Network Parameter Design

The detailed configuration of each layer in the proposed CA-WTBNet is summarized in Table 1. The network takes a five-channel frontend representation as input, and the input channel attention module adopts a reduction ratio of 2. The encoder consists of three convolutional stages with output channels of 32, 64, and 96, respectively. The corresponding convolutional kernel sizes are 64, 32, and 16, and each convolutional stage uses max-pooling with a pooling size of 2 for temporal downsampling. Window-based Transformer blocks are inserted after the second and third encoder stages to enhance temporal dependency modeling. The bottleneck module contains a multi-resolution convolutional branch with kernel sizes of {3,5,9,15}, followed by bottleneck attention for high-level feature enhancement. The decoder contains three transposed-convolution stages, which progressively restore the temporal resolution with the aid of skip connections from the encoder. The temporal modeling module uses a two-layer bidirectional LSTM with 24 hidden units in each direction, followed by temporal attention and three fully connected layers with output dimensions of 24, 8, and 1, respectively. The final output corresponds to a single-channel reconstructed ECG signal. The total number of trainable parameters is 1,033,833. The network is trained using the AdamW optimizer for 150 epochs with a batch size of 128. The initial learning rate is set to 0.003, and a cosine annealing schedule is adopted to gradually decay the learning rate during training. The training data are shuffled before each epoch.

## 5. Experimental Validation and Results

### 5.1. Performance Metrics

The reconstruction accuracy of the ECG signals is evaluated using the Pearson correlation coefficient (PCC), normalized root mean squared error (NRMSE), R-peak F1 score, and R-peak timing error. PCC and NRMSE are used to assess waveform-level reconstruction quality, whereas R-peak F1 score and R-peak timing error are used to further evaluate R-peak-level reconstruction performance of the reconstructed ECG signals.

The first metric, PCC, is used to quantify the linear correlation between the radar-reconstructed ECG and the reference ECG. It is computed according to Equation (32):

(32) $$\mathrm{PCC}=\frac{\sum_{i=1}^{L}\left({\hat{y}}_{i}-\bar{\hat{y}}\right)\left(y_{i}-\bar{y}\right)}{\sqrt{\sum_{i=1}^{L}{\left({\hat{y}}_{i}-\bar{\hat{y}}\right)}^{2}}\sqrt{\sum_{i=1}^{L}{\left(y_{i}-\bar{y}\right)}^{2}}}$$

where *L* is the signal length, ${\hat{y}}_{i}$ and $\bar{\hat{y}}$ are the amplitude of the *i*-th sample and the mean value of the reconstructed ECG signal, respectively, and $y_{i}$ and $\bar{y}$ are the corresponding values for the reference ECG signal. A PCC value closer to 1 indicates a stronger correlation and better reconstruction performance.

The second metric, NRMSE, is used to quantify the normalized amplitude discrepancy between the reconstructed ECG and the reference ECG. It reduces the influence of amplitude-range differences among ECG segments and is computed according to Equation (33):

(33) $$\mathrm{NRMSE}=\frac{\sqrt{\frac{1}{L}\sum_{i=1}^{L}{\left({\hat{y}}_{i}-y_{i}\right)}^{2}}}{\max\left(y\right)-\min\left(y\right)}$$

where max(y) and min(y) denote the maximum and minimum amplitudes of the reference ECG signal, respectively. A smaller NRMSE value indicates a lower normalized reconstruction error.

In addition to waveform-level metrics, R-peak-based metrics are introduced to evaluate whether key R-peak characteristics of the ECG waveform are accurately preserved. To calculate these metrics, both the reconstructed ECG and the reference ECG were first resampled from the original sampling rate of 200 Hz to 2000 Hz to reduce timing quantization errors. The open-source physiological signal processing toolbox NeuroKit2 [41] was then used to detect R-peak locations in both the reference ECG and the reconstructed ECG signals. A reconstructed R peak was regarded as correctly detected if it could be matched to a reference R peak within a predefined temporal tolerance window of 50 ms. Based on the matched and unmatched R peaks, the R-peak F1 score was used to evaluate the consistency of R-peak detection, while the R-peak timing error was used to quantify the temporal deviation between matched R peaks.

The R-peak F1 score is calculated as Equation (34):

(34) $$F1_{\mathrm{Rpeak}}=\frac{2\mathrm{TP}}{2\mathrm{TP}+\mathrm{FP}+\mathrm{FN}}$$

where TP, FP, and FN denote the numbers of true-positive, false-positive, and false-negative R peaks, respectively. A higher $F1_{\mathrm{Rpeak}}$ value indicates more consistent R-peak detection between the reconstructed and reference ECG signals.

The R-peak timing error is used to quantify the temporal deviation between matched R peaks in the reconstructed ECG and the reference ECG. Suppose that *M* pairs of R peaks are successfully matched. Let ${\hat{r}}_{m}$ and $r_{m}$ denote the sample indices of the *m*-th matched R peak in the reconstructed and reference ECG signals, respectively, and let $f_{s}$ denote the sampling frequency. The R-peak timing error is calculated as Equation (35):

(35) $$E_{\mathrm{Rpeak}}=\frac{1}{M}\sum_{m=1}^{M}\frac{\left|{\hat{r}}_{m}-r_{m}\right|}{f_{s}}\times 1000\;\mathrm{ms}.$$

A smaller $E_{\mathrm{Rpeak}}$ value indicates more accurate temporal localization of matched R peaks.

For all metrics, the results are calculated over all test segments. In addition to the mean values, the 95% confidence intervals are also reported to provide a more reliable statistical evaluation of the reconstruction performance.

### 5.2. Dataset Description

The dataset used in this study is a publicly available clinical vital-sign dataset containing both radar and ECG signals, provided by Schellenberger et al. [23]. The data were acquired by medical staff from the Department of Palliative Medicine, University Hospital Erlangen. A total of 30 subjects were included, comprising 14 males and 16 females, all in good health condition, with a mean age of 30.7±9.9 years and a mean body mass index of $23.2\pm 3.3\;\mathrm{kg}/m^{2}$. For each subject, the dataset includes recordings under five different conditions, including stationary and non-stationary scenarios. In this study, only the resting condition was selected for experimental analysis to evaluate ECG reconstruction under a stable and controlled physiological state.

**Figure 7:**
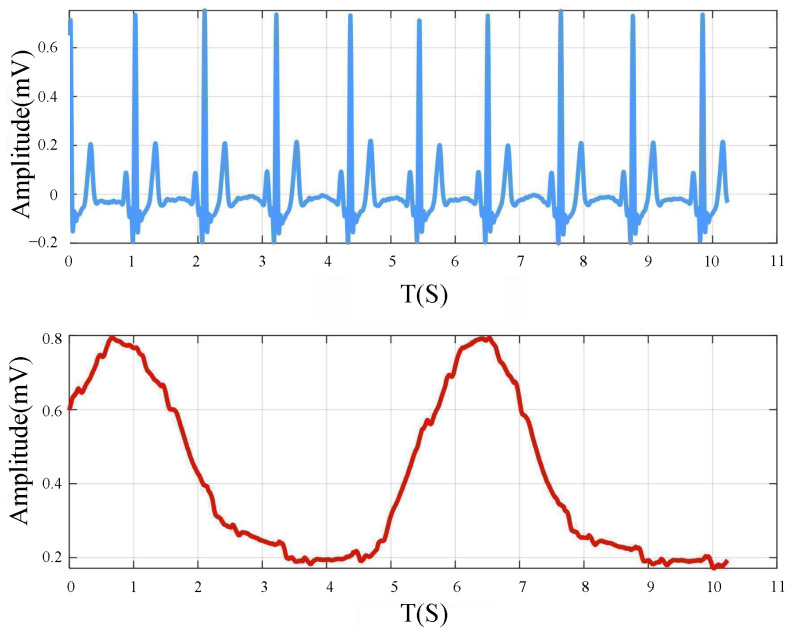
Example ECG and radar signals from the dataset under Resting scenarios.

During data acquisition, each subject lay on an adjustable bed, while a 24GHz continuous-wave radar was positioned 40cm directly above the chest. Simultaneously, ECG signals were synchronously recorded using an adhesive electrode-based ECG acquisition device. The radar and ECG signals were originally sampled at 2000Hz and 1000Hz, respectively. For each subject, continuous radar recordings and synchronized continuous ECG recordings were acquired under the performed scenarios, yielding a total recording duration of approximately 40–60min per subject. The total duration of the entire dataset was approximately 24h. In the present experiments, both signals were first resampled to 200Hz. After resampling, the first 4096 points of each radar and ECG recording were discarded. The remaining synchronized radar and ECG signals were then segmented into non-overlapping segments of 2048 points. Accordingly, the segment length and stride were both set to 2048 points, with no overlap applied. Any remaining samples shorter than 2048 points at the end of a recording were discarded. The synchronously acquired radar and ECG signals after segmentation and preprocessing are shown in Figure 7. Following preprocessing, the radar signal was normalized to the range [0,1] using min–max normalization, whereas the ECG signal was left unnormalized. The dataset included data from 30 subjects in total. To ensure subject-independent evaluation, the subjects were divided into the training and testing sets at a ratio of 5:1. Specifically, data from 25 subjects were used for training and validation, while data from the remaining 5 subjects were used for testing. The number of test samples for each subject is summarized in Table 2. Among the training subjects, 85% of the data were randomly selected for model training, and the remaining 15% were used for validation.

### 5.3. Results of the Proposed Method

#### 5.3.1. Experimental Results of the Proposed Method

**Figure 8:**
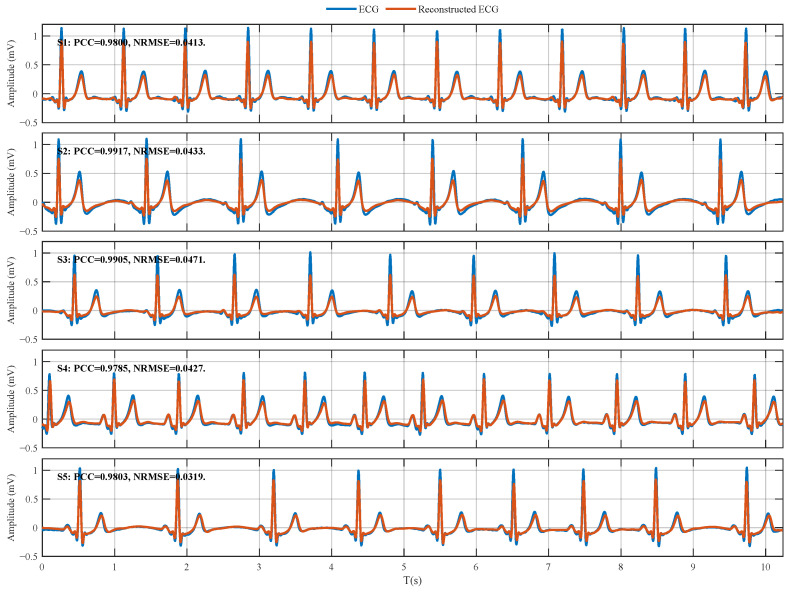
Representative reconstructed ECG examples from the five testing subjects under the resting condition, with one segment selected from each subject for visualization.

**Figure 9:**
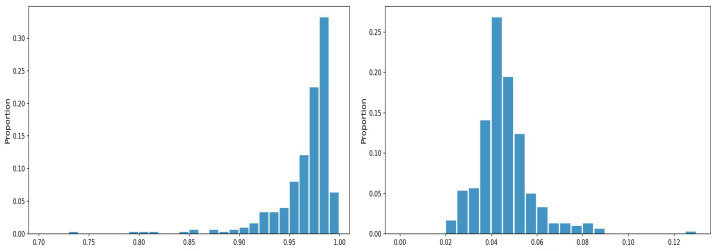
Histograms of the PCC and NRMSE distributions for ECG reconstruction results. The left panel shows the PCC distribution (mean = 0.9641) and the right panel shows the NRMSE distribution (mean = 0.0458).

Representative ECG reconstruction examples from the five testing subjects are further shown in Figure 8. The histogram of the PCC values obtained by the proposed method on the test set is shown in the left panel of Figure 9, with a mean of 0.9641 and a standard deviation of 0.0421. Most PCC values are concentrated in the interval [0.9,1]. The histogram of the NRMSE values is shown in the right panel of Figure 9, with a mean of 0.0458 and a standard deviation of 0.0122. Most NRMSE values are concentrated in the interval [0.02,0.08]. The high PCC mean values, low NRMSE mean values, and relatively limited overall dispersion suggest generally favorable and relatively consistent reconstruction performance on the test set. These pooled segment-level PCC and NRMSE results further indicate that the proposed method achieves high reconstruction accuracy.

**Figure 10:**
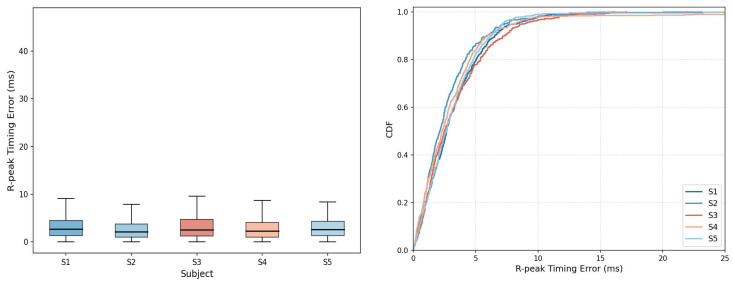
R-peak timing error analysis for ECG reconstruction results. The left panel shows the boxplot distribution of R-peak timing errors across the five testing subjects after outlier removal, and the right panel shows the CDF of R-peak timing errors.

Figure 10 shows the boxplot and cumulative distribution function (CDF) curves of R-peak timing errors for the five testing subjects. As shown by the CDF curves, the distributions across the five subjects are highly consistent, and approximately 90% of the R-peak timing errors are below 7.5 ms. This indicates that the proposed method achieves accurate and stable R-peak temporal localization across different subjects. The boxplot further shows that most R-peak timing errors are concentrated within the range of 0–5 ms, with median values mostly around 2–3 ms for the five subjects. In detail, the median R-peak errors for S1–S5 were 2.66, 2.14, 2.50, 2.29, and 2.63 ms, respectively, with an overall median error of 2.47 ms. These results demonstrate that the reconstructed ECG signals can preserve heartbeat event timing with good temporal consistency.

Table 3 summarizes the subject-wise reconstruction performance in terms of PCC, NRMSE, R-peak F1 score, and R-peak timing error, while Table 4 reports the subject-level 95% confidence intervals for these metrics. To account for within-subject dependency, each metric was first averaged within each held-out subject, and the confidence intervals were then calculated from the five subject-level mean values. For waveform-level evaluation, the mean PCC values of the five testing subjects range from 0.9573 to 0.9737, while all NRMSE values are below 0.0503. In addition, the relatively narrow subject-level confidence intervals of PCC and NRMSE suggest that the waveform-level performance was reasonably stable across the five held-out subjects. These results suggest that the reconstructed ECG signals maintain high morphological similarity and low reconstruction error compared with the reference ECG signals.

For heartbeat-level evaluation, the R-peak F1 scores are above 0.99 for all testing subjects, with confidence intervals close to 1. The mean R-peak timing errors vary only slightly across subjects, ranging from 2.72 ms to 3.37 ms. Overall, the proposed method achieves a mean R-peak timing error of 3.13 ms, with a 95% confidence interval of [2.83, 3.44] ms. These results indicate that the proposed method can reliably preserve heartbeat events and accurately localize R peaks in the reconstructed ECG signals. Overall, the results demonstrate the stable cross-subject reconstruction performance of the proposed method.

#### 5.3.2. Comparative Experimental Results

To further evaluate the effectiveness of the proposed method, we compared CA-WTBNet with several existing ECG reconstruction approaches, as summarized in Table 5. CNN-LSTM [12], AM-CNN-GRU [20], and CAE-BiLSTM [13] were re-implemented using the same dataset partitioning and evaluation protocol as the proposed method, providing a controlled comparison under identical experimental conditions. Additionally, the reported performance of RSSRnet [14], evaluated based on the resting state of the same public dataset, is included for reference. The results indicate that the proposed network achieves higher PCC values and lower NRMSE compared with the reproduced baseline methods. More specifically, relative to the best-performing reference method, RSSRnet, CA-WTBNet improves PCC by approximately 0.53% and reduces NRMSE by approximately 10.20%. These findings demonstrate that the proposed method effectively captures heartbeat-related information and more robustly models the complementary relationships among multi-channel input features, local heartbeat waveform patterns, and long-range temporal dependencies, thereby achieving high-accuracy ECG waveform reconstruction under the test conditions.

#### 5.3.3. Ablation Experimental Results

To further investigate the contribution of each key component and validate the rationality of the proposed design, a series of ablation experiments were conducted. The ablation study consists of three parts: network-structure ablation, signal-processing frontend ablation, and loss-function ablation. For each experiment, one component was removed while the remaining experimental settings were kept unchanged, resulting in several model variants being available for comparison.

The quantitative results of the network-structure ablation are summarized in Table 6. Compared with the complete network, removing any major component leads to performance degradation, indicating that each module contributes positively to feature extraction and signal reconstruction. Among these components, the encoder Transformer module and the deep residual convolutional module have the most significant influence. After removing the encoder Transformer module, the PCC decreases from 0.9641 to 0.9488, and the R-peak timing error increases from 3.13 ms to 6.09 ms. This suggests that the window-based self-attention mechanism is important for modeling long-range heartbeat dependencies and local waveform context. The deep residual structure also plays an important role in stabilizing feature propagation. When it is removed, both waveform-level metrics and R-peak-based metrics deteriorate noticeably. In addition, the channel-attention modules are also effective. ECA attention recalibrates feature channels within the encoder, whereas input channel attention adaptively weights the five frontend input channels. The degradation observed after removing these two modules further confirms their contribution to multi-channel feature fusion.

Table 7 presents the ablation results of the signal-processing frontend. Compared with using the raw input only, the complete frontend achieves better performance across all evaluation metrics. Specifically, the PCC increases from 0.9530 to 0.9641, the NRMSE decreases from 0.0549 to 0.0458, the R-peak F1 score improves from 0.9736 to 0.9956, and the R-peak timing error decreases from 4.84 ms to 3.13 ms. These results indicate that the proposed frontend provides more effective heartbeat-related representations than the raw radar signal, thereby improving both waveform-level reconstruction accuracy and heartbeat-event-level temporal localization. Among the input channels, removing the $x_{\mathrm{bp}}$ channel causes the most significant performance degradation. The PCC decreases from 0.9641 to 0.9101, the NRMSE increases from 0.0458 to 0.0651, and the R-peak timing error increases from 3.13 ms to 5.40 ms. This demonstrates that the bandpass heartbeat-related channel plays an important role in preserving ECG-relevant temporal information. In addition, removing the respiratory suppression module also leads to degradation in all metrics. The PCC decreases from 0.9641 to 0.9583, the NRMSE increases from 0.0458 to 0.0495, the R-peak F1 score decreases from 0.9956 to 0.9922, and the R-peak timing error increases from 3.13 ms to 3.40 ms. These results suggest that respiratory harmonic suppression helps to reduce the influence of respiratory interference on heartbeat-related components and further stabilizes R-peak temporal localization in the reconstructed ECG.

Table 8 shows the ablation results of the loss function. The complete objective consists of Huber loss, PCC loss, frequency-domain loss, and multi-scale PCC loss. Removing any individual loss term causes varying degrees of performance degradation, indicating that these loss components are complementary and jointly provide more comprehensive supervision for ECG reconstruction. The PCC loss and the multi-scale PCC loss have relatively clear effects on the overall performance. Specifically, after removing the PCC loss, the PCC decreases to 0.9539, the NRMSE increases to 0.0498, and the R-peak timing error increases to 4.23 ms. This indicates that the PCC loss is important for maintaining the overall waveform correlation between the reconstructed ECG and the reference ECG. In addition, the frequency-domain loss mainly constrains spectral consistency. Although removing it only slightly affects the PCC, it increases the NRMSE and R-peak timing error, suggesting that frequency-domain supervision helps reduce amplitude-structure distortion and improves the stability of R-peak-related morphology.

Overall, the ablation results confirm the effectiveness of the proposed network architecture, signal-processing frontend, and multi-objective loss function.

## 6. Discussion and Open Research Questions

### 6.1. Discussion

The current research develops an entire process from CW radar signal pre-processing, via the respiration-suppressing front-end, to the ECG reconstruction network. Experiments on an open data set show good effectiveness, providing a feasible method to solve the problems mentioned in the statement. The results show that the proposed front-end, which includes raw heartbeat components, respiration-suppressed components, and relevant input channels, can efficiently reduce the interference of respiration while retaining the important cardiac information. Ablation tests on the front-end confirm this conclusion: compared with using only the raw radar signal or the suppressed component, the integrated representation has more heartbeat-related features. In the CA-WTBNet back-end, multi-channel information, local waveform patterns, and temporal dependencies are jointly considered to further improve the ECG reconstruction performance. In particular, the input channel attention and ECA modules improve the selection of channel-level features so that the network can pay more attention to the slight cardiac signals. The convolutional and residual blocks are able to capture the local waveform morphology, such as the peaks and inflection points that stabilize the transmission of heartbeat features. Meanwhile, the window-based Transformer and BiLSTM modules represent the long-range and bidirectional temporal dependencies, respectively, guaranteeing the overall periodicity of the heartbeat and avoiding rhythm distortion and temporal errors in the reconstructed signal. The results of the ablation test indicate that if any of these components, especially the encoder Transformer and deep residual blocks, are removed, there will be a considerable decrease in performance.

From a clinical and practical perspective, the proposed method may be relevant in situations where conventional electrode-based ECG monitoring is inconvenient, poorly tolerated, or difficult to maintain continuously. A representative example is controlled single-person resting monitoring, in which a millimeter-wave radar may be positioned above the chest to provide low-burden cardiac monitoring without direct skin contact. Such a setting may be of particular interest in situations where prolonged electrode attachment is undesirable or impractical. Nevertheless, this potential clinical and practical value should be interpreted cautiously, since the present study was validated only on healthy subjects under controlled resting conditions.

### 6.2. Open Research Questions

Several limitations should be noted in the present study. First, even though the testing set did not include the same subjects as the training set, the number of testing subjects was small. More validation is needed with larger and more diverse groups. Second, this study only looked at the resting condition, so it is still unclear how well the method works when body movement, irregular breathing, or health problems are present. Third, the current evaluation focused on waveform-level metrics and R-peak-based analysis. In the future, we plan to look at more detailed clinical interval measurements, such as PR, QRS, and QT intervals, once we can more reliably identify the start and end points of the P-wave, QRS complex, and T-wave. Finally, before this method can be used for clinical diagnosis, it should be tested further on ECG recordings from patients with clinical abnormalities.

## 7. Conclusions

This study proposes a millimeter-wave radar-based ECG reconstruction method that integrates a multi-channel frontend with respiratory-harmonic suppression and the CA-WTBNet network. The proposed method outperformed the existing heartbeat-component extraction methods and reconstruction networks under the tested conditions, with a mean PCC of 0.9641 (standard deviation of 0.0421), a mean NRMSE of 0.0458 (standard deviation of 0.0122), an average R-peak F1 score of 0.9956, and a mean R-peak timing error of 3.13 ms. The results show that the proposed method is more effective at extracting heartbeat-related information and at jointly modeling multi-channel complementary features, local waveform patterns, and temporal dependencies, thereby improving the performance of ECG reconstruction. Overall, the results demonstrate the potential of the proposed method for non-contact reconstruction of ECG from millimeter-wave radar signals in healthy subjects at rest.

## Figures and Tables

**Table 1:** Layer-wise configuration of the proposed CA-WTBNet. Boldface in the final row indicates the total number of trainable parameters.

Stage	Layer	Output Shape	Kernel/Config	Params	Activation
Input	Input Channel Attention	5×2048	SE reduction =2	27	ReLU + Sigmoid
	AdaptiveAvgPool1d	5×1	–	0	–
	Linear (5→2)	2	–	12	ReLU
	Linear (2→5)	5	–	15	Sigmoid
Encoder	ConvBlock 1	32×1024	K=64, S=1, P=same + MaxPool K=2	10,339	Tanh
	SamePadConv1d (5→32)	32×1024	K=64	10,272	–
	BatchNorm1d + ECA	32×1024	γ=2, b=1	67	Sigmoid
	ResidualConvBlock 1	32×1024	K=7, D=1	14,528	Tanh
	ConvBlock 2	64×512	K=32, S=1, P=same + MaxPool K=2	65,731	Tanh
	ResidualConvBlock 2	64×512	K=5, D=1	41,344	Tanh
	TransformerBlock (enc2)	64×512	Heads =4, Win =64	33,474	GELU
	WindowAttention	64×512	Heads =4, Win =64	16,769	–
	FFN (64→128→64)	64×512	Ratio =2.0	16,576	GELU
	ConvBlock 3	96×256	K=16, S=1, P=same + MaxPool K=2	98,595	Tanh
	ResidualConvBlock 3	96×256	K=3, D=1	55,872	Tanh
	TransformerBlock (enc3)	96×256	Heads =4, Win =64	74,786	GELU
Bottleneck	MultiResolution Branch	96×256	K∈{3,5,9,15}	83,520	Tanh
	4 branches + 1×1 mix	96×256	C/4 per branch	–	–
	BottleneckAttention	96×256	Heads =4, Dropout =0.05	37,440	–
	MultiheadAttention + LayerNorm	96×256	–	–	–
Decoder	TransConvBlock 1	96×512	ConvTranspose K=16, S=2	147,744	Tanh
	Skip Conv3 (96→96)	96×512	1×1 Conv	9312	–
	TransConvBlock 2	64×1024	ConvTranspose K=32, S=2	196,800	Tanh
	Skip Conv2 (64→64)	64×1024	1×1 Conv	4160	–
	TransConvBlock 3	32×2048	ConvTranspose K=64, S=2	131,168	Tanh
	Skip Conv1 (32→32)	32×2048	1×1 Conv	1056	–
Temporal	Bi-LSTM (2 layers)	2048×48	Hidden =24, Bidirectional	25,344	–
	Layer 0 (forward + reverse)	2048×48	Input =32, Hidden =24	11,136	–
	Layer 1 (forward + reverse)	2048×48	Input =48, Hidden =24	14,208	–
	Temporal Attention	2048×48	48→24→1	1201	Tanh + Sigmoid
	FC1 (48→24)	2048×24	–	1176	Tanh
	FC2 (24→8)	2048×8	–	200	Tanh
	FC3 (8→1)	2048×1	–	9	–
Output	Residual Skip Projection	1×2048	Conv1d (5→1,K=1) on input	6	–
	Residual Scale	scalar	learnable, init =0.1	1	–
	output = FC_out +λ·proj(x_input)	–	–	–	–
**Total**				**1,033,833**	–

**Table 2:** Number and proportion of test samples for each subject.

Subject	S1	S2	S3	S4	S5	Overall
Number of samples *n*	58	62	59	60	59	298
Proportion (%)	19.46	20.81	19.80	20.13	19.80	100.00

**Table 3:** Per-subject segment-level reconstruction performance on the testing set (mean ± std).

Subject	PCC	NRMSE	R-Peak F1	R-Peak Error (ms)
S1	0.9642 ± 0.0296	0.0479 ± 0.0106	0.9979 ± 0.0092	3.21 ± 1.46
S2	0.9737 ± 0.0294	0.0476 ± 0.0082	0.9980 ± 0.0112	2.72 ± 1.67
S3	0.9587 ± 0.0451	0.0503 ± 0.0089	0.9912 ± 0.0278	3.37 ± 1.96
S4	0.9573 ± 0.0649	0.0484 ± 0.0159	0.9927 ± 0.0277	3.24 ± 2.55
S5	0.9663 ± 0.0284	0.0348 ± 0.0091	0.9981 ± 0.0102	3.14 ± 1.67
**Overall**	0.9641 ± 0.0421	0.0458 ± 0.0122	0.9956 ± 0.0194	3.13 ± 1.90

Note: Boldface identifies the pooled Overall result. The Overall row reports pooled segment-level descriptive statistics across all testing segments.

**Table 4:** Subject-level 95% confidence intervals for PCC, NRMSE, R-peak F1 score, and R-peak timing error on the testing set.

	PCC	NRMSE	R-Peak F1	R-Peak Error (ms)
Subject-level 95% CI	[0.9559,0.9722]	[0.0381,0.0535]	[0.9914,0.9997]	[2.83,3.44]

Note: The 95% confidence intervals were calculated from the five subject-level mean values to account for within-subject dependency.

**Table 5:** Comparison with existing ECG reconstruction methods based on PCC and NRMSE. ↑ and ↓ indicate that higher and lower values are better, respectively. Boldface highlights the proposed method.

Model	PCC ↑	NRMSE ↓
CNN-LSTM	0.8884±0.0808	0.0771±0.0168
AM-CNN-GRU	0.9231±0.0735	0.0665±0.0153
CAE-BiLSTM	0.9291±0.0615	0.0626±0.0141
RSSRnet	0.9590±0.0390	0.0510±0.0220
**CA-WTBNet (Ours)**	0.9641±0.0421	0.0458±0.0122

**Table 6:** Network structure ablation results of the proposed model. ↑ and ↓ indicate that higher and lower values are better, respectively.

Configuration	PCC ↑	NRMSE ↓	R-Peak F1 ↑	R-Peak Err. (ms) ↓
Model_medium (Baseline)	0.9641	0.0458	0.9956	3.13
w/o ECA Attention	0.9513	0.0604	0.9751	5.58
w/o Deep Residual	0.9442	0.0607	0.9706	5.82
w/o Skip Connection	0.9621	0.0524	0.9897	3.67
w/o Residual Skip	0.9612	0.0558	0.9862	3.98
w/o Temporal Attention	0.9579	0.0587	0.9828	4.21
w/o Multi-Resolution	0.9593	0.0545	0.9847	4.05
w/o Input Ch. Attn.	0.9539	0.0549	0.9793	4.31
w/o Bottleneck Attn.	0.9541	0.0531	0.9806	4.18
w/o Encoder Transformer	0.9488	0.0619	0.9674	6.09

**Table 7:** Signal processing frontend ablation results of the proposed model. ↑ and ↓ indicate that higher and lower values are better, respectively.

Configuration	PCC ↑	NRMSE ↓	R-Peak F1 ↑	R-Peak Err. (ms) ↓
Model_medium (Baseline)	0.9641	0.0458	0.9956	3.13
Raw Only	0.9530	0.0549	0.9736	4.84
w/o Suppression	0.9583	0.0495	0.9922	3.40
w/o $x_{\mathrm{bp}}$ Channel	0.9101	0.0651	0.9756	5.40
w/o $f_{\mathrm{card}}$ Protection	0.9615	0.0439	0.9923	3.30

**Table 8:** Loss function ablation results of the proposed model. ↑ and ↓ indicate that higher and lower values are better, respectively.

Configuration	PCC ↑	NRMSE ↓	R-Peak F1 ↑	R-Peak Err. (ms) ↓
Full loss (Baseline)	0.9641	0.0458	0.9956	3.13
w/o PCC Loss	0.9539	0.0498	0.9827	4.23
w/o Freq Loss	0.9638	0.0517	0.9918	3.45
w/o Multi-scale PCC	0.9542	0.0526	0.9819	4.35

## Data Availability

The data analyzed in this study are publicly available from the radar vital-sign dataset with synchronized ECG recordings released by Schellenberger et al. No new data were created in this study.

## References

[1] Roth G.A., Johnson C.O., Abate K.H., Abd-Allah F., Ahmed M., Alam K., Alam T., Alvis-Guzman N., Ansari H., Ärnlöv J. (2018). The burden of cardiovascular diseases among US states, 1990–2016. JAMA Cardiol..

[2] Jing S.F. (2022). Analysis of remote dynamic electrocardiogram monitoring results in arrhythmia patients of different age groups. J. Pract. Med. Tech..

[3] Xu S., Rwei A., Vwalika B., Chisembele M.P., Stringer J.S.A., Ginsburg A.S., Rogers J.A. (2021). Wireless skin sensors for physiological monitoring of infants in low-income and middle-income countries. Lancet Digit. Health.

[4] Bloe C. (2021). The role of single-use ECG leads in reducing healthcare-associated infections. Br. J. Nurs..

[5] Marsh D.F., Whitehead N.J., Meiklereid N.S., Smith G.B. (1993). ECG monitoring in severely burned patients–a simple solution!. Burns.

[6] Gao Z., Ali L., Wang C., Liu R., Wang C., Qian C., Sung H., Meng F. (2022). Real-time non-contact millimeter-wave radar-based vital sign detection. Sensors.

[7] Paterniani G., Sgreccia D., Davoli A., Guerzoni G., Di Viesti P., Valenti A.C., Vitolo M., Vitetta G.M., Boriani G. (2023). Radar-Based Monitoring of Vital Signs: A Tutorial Overview. Proc. IEEE.

[8] Zhang X., Liu C., Cheng Y., Li Z., Xu C., Huang C., Zhan Y., Bo W., Xia J., Xu W. (2025). A comprehensive survey of research trends in mmWave technologies for medical applications. Sensors.

[9] Zhang B.-B., Zhang D., Li Y., Lu Z., Chen J., Wang H., Zhou F., Pu Y., Hu Y., Ma L.-K. (2024). Monitoring long-term cardiac activity with contactless radio frequency signals. Nat. Commun..

[10] Yang Y.L., Liang C.H., Feng Y.F., Liu G.S., He Y. (2023). Research status and prospects of non-contact vital sign monitoring technology. China Med. Devices.

[11] Michler F., Shi K., Schellenberger S., Steigleder T., Malessa A., Hameyer L., Neumann N., Lurz F., Ostgathe C., Weigel R. (2019). A Clinically Evaluated Interferometric Continuous-Wave Radar System for the Contactless Measurement of Human Vital Parameters. Sensors.

[12] Yamamoto K., Hiromatsu R., Ohtsuki T. (2020). ECG signal reconstruction via Doppler sensor by hybrid deep learning model with CNN and LSTM. IEEE Access.

[13] Duan W., Huang T.L., Liu J., Hang X.Y. (2024). Study on an attention mechanism-based millimeter-wave radar ECG signal inversion algorithm. Guangxi Sci..

[14] Luo J.X., Zhang Y.H., Dai X., Fu D., Liu K. (2024). A non-contact electrocardiogram reconstruction algorithm based on millimeter-wave radar. Telecommun. Sci..

[15] Xu D., Xu Y., Xu K., Hu Z., Xing M., Gini F., Greco M.S. (2025). WaveGRU-Net: Robust Non-Contact ECG Reconstruction via MIMO Millimeter-Wave Radar and Multi-Scale Semantic Analysis. Signal Process..

[16] Ji S., Zhang Z., Xia Z., Wen H., Zhu J., Zhao K. (2022). RBHHM: A Novel Remote Cardiac Cycle Detection Model Based on Heartbeat Harmonics. Biomed. Signal Process. Control.

[17] Xiong Y., Peng Z., Gu C., Li S., Wang D., Zhang W. (2020). Differential Enhancement Method for Robust and Accurate Heart Rate Monitoring via Microwave Vital Sign Sensing. IEEE Trans. Instrum. Meas..

[18] Yu R., Bliss D.W. (2019). Remote Sensing for Vital Information Based on Spectral-Domain Harmonic Signatures. IEEE Trans. Aerosp. Electron. Syst..

[19] Ding Y., Yu X., Lei C., Sun Y., Xu X., Zhang J. (2020). A Novel Real-Time Human Heart Rate Estimation Method for Noncontact Vital Sign Radar Detection. IEEE Access.

[20] Wu Y., Ni H., Mao C., Han J. (2024). Contactless reconstruction of ECG and respiration signals with mmWave radar based on RSSRnet. IEEE Sens. J..

[21] Zhang Y., Yang R., Yue Y., Lim E.G. (2025). radarODE-MTL: A multitask learning framework with eccentric gradient alignment for robust radar-based ECG reconstruction. IEEE Trans. Instrum. Meas..

[22] Wang Z., Jin B., Li S., Zhang F., Zhang W. (2023). ECG-grained Cardiac Monitoring Using UWB Signals. Proc. ACM Interact. Mob. Wearable Ubiquitous Technol..

[23] Schellenberger S., Shi K., Steigleder T., Malessa A., Michler F., Hameyer L., Neumann N., Lurz F., Weigel R., Ostgathe C. (2020). A dataset of clinically recorded radar vital signs with synchronised reference sensor signals. Sci. Data.

[24] Will C., Shi K., Schellenberger S., Steigleder T., Michler F., Fuchs J., Weigel R., Ostgathe C., Koelpin A. (2018). Radar-Based Heart Sound Detection. Sci. Rep..

[25] Singh A., Gao X., Yavari E., Zakrzewski M., Cao X.H., Lubecke V.M., Boric-Lubecke O. (2013). Data-based quadrature imbalance compensation for a CW Doppler radar system. IEEE Trans. Microw. Theory Tech..

[26] Park B.-K., Boric-Lubecke O., Lubecke V.M. (2007). Arctangent Demodulation With DC Offset Compensation in Quadrature Doppler Radar Receiver Systems. IEEE Trans. Microw. Theory Tech..

[27] Han K., Hong S. (2020). Phase-extraction method with multiple frequencies of FMCW radar for human body motion tracking. IEEE Microw. Wirel. Compon. Lett..

[28] Mallat S.G. (1989). A theory for multiresolution signal decomposition: The wavelet representation. IEEE Trans. Pattern Anal. Mach. Intell..

[29] Dragomiretskiy K., Zosso D. (2014). Variational mode decomposition. IEEE Trans. Signal Process..

[30] Percival D.B., Walden A.T. (2000). Wavelet Methods for Time Series Analysis.

[31] Awal M.A., Mostafa S.S., Ahmad M., Rashid M.A. (2014). An adaptive level dependent wavelet thresholding for ECG denoising. Biocybern. Biomed. Eng..

[32] Wang L., Sun W., Chen Y., Li P., Zhao L. Wavelet Transform Based ECG Denoising Using Adaptive Thresholding. Proceedings of the 7th International Conference on Bioinformatics and Biomedical Science (ICBBS).

[33] Hu J., Shen L., Sun G. (2018). Squeeze-and-excitation networks. Proceedings of the IEEE/CVF Conference on Computer Vision and Pattern Recognition.

[34] Krizhevsky A., Sutskever I., Hinton G.E. (2017). ImageNet classification with deep convolutional neural networks. Commun. ACM.

[35] Ioffe S., Szegedy C. (2015). Batch normalization: Accelerating deep network training by reducing internal covariate shift. Proceedings of the International Conference on Machine Learning.

[36] Wang Q., Wu B., Zhu P., Li P., Zuo W., Hu Q. (2020). ECA-Net: Efficient channel attention for deep convolutional neural networks. Proceedings of the IEEE/CVF Conference on Computer Vision and Pattern Recognition.

[37] Liu Z., Lin Y., Cao Y., Hu H., Wei Y., Zhang Z., Lin S., Guo B. (2021). Swin Transformer: Hierarchical Vision Transformer using Shifted Windows. Proceedings of the IEEE/CVF International Conference on Computer Vision (ICCV).

[38] Yildirim O. (2018). A novel wavelet sequence based on deep bidirectional LSTM network model for ECG signal classification. Comput. Biol. Med..

[39] Hochreiter S., Schmidhuber J. (1997). Long short-term memory. Neural Comput..

[40] Graves A., Schmidhuber J. (2005). Framewise Phoneme Classification with Bidirectional LSTM and Other Neural Network Architectures. Neural Netw..

[41] Makowski D., Pham T., Lau Z.J., Brammer J.C., Lespinasse F., Pham H., Schölzel C., Chen S.H.A. (2021). NeuroKit2: A Python toolbox for neurophysiological signal processing. Behav. Res. Methods.

